# Prioritising, Ranking and Resource Implementation - A Normative Analysis

**DOI:** 10.15171/ijhpm.2017.125

**Published:** 2017-10-29

**Authors:** Lars Sandman

**Affiliations:** ^1^National Center for Priority Setting in Health-Care, Department of Medicine and Health, Linköping University, Linköping, Sweden.; ^2^Academy for Care, Worklife and Welfare, University of Borås, Borås, Sweden.

**Keywords:** Priority Setting, Ethics, Ranking, Reimbursement

## Abstract

**Background:** Priority setting in publicly financed healthcare systems should be guided by ethical norms and other considerations viewed as socially valuable, and we find several different approaches for how such norms and considerations guide priorities in healthcare decision-making. Common to many of these approaches is that interventions are ranked in relation to each other, following the application of these norms and considerations, and that this ranking list is then translated into a coverage scheme. In the literature we find at least two different views on how a ranking list should be translated into coverage schemes: (1) rationing from the bottom where everything below a certain ranking order is rationed; or (2) a relative degree of coverage, where higher ranked interventions are given a relatively larger share of resources than lower ranked interventions according to some "curve of coverage."

**Methods:** The aim of this article is to provide a normative analysis of how the background set of ethical norms and other considerations support these two views.

**Results:** The result of the analysis shows that rationing from the bottom generally gets stronger support if taking background ethical norms seriously, and with regard to the extent the ranking succeeds in realising these norms. However, in non-ideal rankings and to handle variations at individual patient level, there is support for relative coverage at the borderline of what could be covered. A more general relative coverage curve could also be supported if there is a need to generate resources for the healthcare system, by getting patients back into production and getting acceptance for priority setting decisions.

**Conclusion:** Hence, different types of reasons support different deviations from rationing from the bottom. And it should be noted that the two latter reasons will imply a cost in terms of not living up to the background set of ethical norms.

## Background

Due to demographic and medical developments, increased patient expectations, and situations of fiscal austerity, healthcare priority setting should top the agenda of most healthcare systems. This is especially true when it concerns healthcare systems in welfare states where there is generally a demand for equitable use of resources, and the distribution of resources is not solely guided by individual patient preferences. But, generally, in any healthcare system with societal coverage, where available resources do not match the existent healthcare needs, priority setting will have to be achieved – be it the prioritisation of innovative and costly interventions in a wealthy country like Sweden, or of interventions essential to public health in rural parts of Africa.^1^

Defining healthcare priority setting, in this context, as the explicit ranking of healthcare interventions to guide resource distribution, implies that priority setting should in turn be guided by a set of relevant considerations. There is great diversity as to what exactly this set should include, even if different suggestions tend to overlap. However, all explicit suggestions include a number of ethical considerations at the core of this set (see for example Persad et al for an overview).^2^ These ethical considerations can be expressed in terms of legally implemented, ethical principles or criteria for priority setting (such as the principles of human dignity, needs-solidarity and cost-effectiveness in the Swedish context,^1^ or the criteria of benefit, resources and severity in the Norwegian context^3,4^), a specific tool including a number of ethically motivated criteria (eg, the use of the multi-criteria analysis tool EVIDEM in Catalonia),^5,6^ a specific process following processual criteria with the aim of creating a beneficial environment for making ethically relevant considerations (eg, following the standards of accountability for reasonableness in priority setting in HIV/AIDS-control in West Java Indonesia etc),^7,8^ or a combination of approaches. Besides the ethical core considerations, the set of considerations might also include references to more pragmatic considerations, such as whether budget impact will allow a specific intervention to be reimbursed, even given a high ranking. New biological drugs for hepatitis C are a current example where a number of ethical considerations support their use, but where budget impact hinders or delays implementation of such use.^9^

Regardless of approach, explicit priority setting should be guided by a set of relevant considerations, with a core of ethical considerations. Let me return to this below. There are different ways to prioritise within healthcare. It can be done by looking at single new technologies and deciding whether they should be prioritised for funding given the relevant considerations, exemplified by when the National Institute for Health and Care Excellence (NICE) in England assess new healthcare interventions and provide clinical guidelines.^10^ Another approach is to look at a *set* of healthcare interventions or condition/intervention-pairs^ [1] ^, and try to translate the relevant considerations into a priority ranking between these different interventions. This is exemplified by the Oregon priority list,^11^ or the Swedish national guidelines within different disease areas.^12^ It is upon this latter approach that I will focus in this article.

When the ranking is done, the resulting ranking list needs to be implemented in a reimbursement or coverage scheme (henceforth coverage scheme) within the healthcare system. Literature presents at least two different ways^ [2]^ of implementing or translating a ranking list into a coverage scheme.^3,11^

The Oregon list was an example of the idea of starting from the top of the list going down, and covering everything until the budget limit is reached. To the extent that available resources do not cover everything on the list, the technologies below the budget line should be rationed^ [3] ^. Another way to express this is to say that we should *ration from the bottom* of the ranking list (RATBOT for short).
The Swedish ethics platform for priority setting and the Swedish national guidelines recommend another approach, here labelled *relative degree of needs coverage* or more generally, *relative resource coverage* (RELCOV for short). This idea implies that the higher the rank, the more relative the coverage, and vice versa for what is lower on the ranking list.

The two ideas will be explained in more detail further into the article.

These ideas on how a ranking list should be translated into resource coverage within the healthcare system, raise a normative question: Are they normatively equivalent or are there normative reasons to prefer one before the other? In previous articles, unacceptable trade-offs in priority setting have been identified,^1^ but no analysis comparing these two systematic methods of translating a ranking list into a coverage scheme is available in the scientific literature. The aim of this article is to analyse whether there are normative reasons to prefer either RATBOT or RELCOV. Before moving into the analysis, let me first elaborate on how a ranking list is created from a set of considerations.

### From a Set of Considerations to a Ranking List

In order to further analyse the normative implications of different coverage schemes, it is important to elaborate on how a ranking list should (ideally) be arrived at given an explicit set of considerations. Regardless of whether a set of explicit principles or criteria is used, or whether we follow a formalised process where the set of considerations arises more dynamically, ideally these considerations should be applied consistently. This implies that there should be a consistent balancing of the different considerations resulting in a consistent ranking of interventions. Obviously, this means that if different interventions get the same values on each consideration, they should result in the same ranking. It also means that if a specific consideration has a certain importance for one intervention, it should also have similar importance for other interventions. To illustrate, if severity is part of our considerations and we claim that great severity is of major importance for giving a high ranking to one intervention, we cannot claim that great severity is of minor importance in relation to another intervention. Of course, severity can be differently balanced taking other considerations into account. If cost-effectiveness is one of the relevant considerations, great severity and low cost-effectiveness can result in a low ranking, but that does not downplay the importance of severity, and another intervention with equally low cost-effectiveness and lower severity should result in an even lower ranking. Generally, in an ideal ranking, we will have applied the set of considerations consistently, and all interventions ranked 1 will then be more important to cover (given our balanced set of considerations), than all interventions ranked 2 etc. This is true, regardless of what the set of considerations we base our ranking on contains. Does this also imply that it is *ethically* more important to cover what is ranked 1 than what is ranked 2, and so on?

As indicated above, even if ethical considerations form the core, the set of priority setting considerations can also include other considerations of a more pragmatic nature. To some extent, this is dependent on how we define ethical considerations. In this context, ethical considerations are all considerations that are related to some value of human life. This implies, for example, that effectiveness and cost-effectiveness considerations are ethical considerations, since they concern whether and how value is distributed to people. The same goes for evidential concerns, eg, whether there is ample evidence of the effectiveness of the intervention etc, since this will affect the potential to benefit, whether use of resources on the intervention risk being wasted, how patients should be able to make an informed decision etc.^13^ If we are uncomfortable using the term ethically important, we might instead prefer the term socially valuable, ie, the set of considerations that should guide priority setting should be a set reflecting what are considered to be socially relevant values in the society or context at hand. If we add more pragmatic concerns to our set of considerations, they should be added because they reflect obstacles to implementing a specific ranking, which at least in the short run is insurmountable, eg, access to available resources such as professional competence. Essentially, this means that what is ranked 1, given an ideal ranking exercise, will also be more socially valuable than what is ranked 2 etc.

### Different Coverage Schemes: RATBOT and RELCOV

Before analysing how different coverage schemes would fare in relation to such an ideal, it is also necessary to describe in more detail the implications of the two versions of coverage above. To illustrate the reasoning in this article let us initially assume that decision-makers are ranking five different interventions (A-E), needed by 100 patients, at the total cost of 100 000 € per intervention. The ranking follows the set of relevant considerations established within the healthcare system at hand. Let us also assume that the available budget amounts to 300 000 €. RATBOT would then imply that interventions A-C are funded and D-E are rationed (Table 1).

**Table 1:** A Ranking of Five Different Interventions Applying Rationing From the Bottom

**Ranking**	**Intervention**	**No. of Patients Getting Treatment**	**Total Cost (€)**
1	A	100	100 000
2	B	100	100 000
3	C	100	100 000
4	D	0	0
5	E	0	0

RELCOV, on the other hand, could follow a number of different coverage curves. Table 2 illustrates a situation in which the ranking from 1-5 is translated into a curve of coverage with the following five steps: 100% - 80% - 60% - 40% - 20%.

**Table 2:** A Ranking of Five Different Interventions Applying a Relative Degree of Coverage

**Ranking**	**Intervention**	**No. of Patients Getting Treatment**	**Total Cost (€)**
1	A	100	100 000
2	B	80	80 000
3	C	60	60 000
4	D	40	40 000
5	E	20	20 000

It could be that instead of patients losing out on the full intervention, it is diluted so that patients in need of intervention B only get 80% thereof (if divisible). If so, it might also be envisioned that patients co-pay the resulting 20% or whatever percentage is outstanding. However, since the focus here is on what should have societal coverage, this possible complication will be ignored. There are obviously a number of possible coverage curves here, and one might claim that RATBOT is a specific version of RELCOV, following a certain coverage curve.

## Methods

In the article, normative analysis following the methodology of reflective equilibrium is used.^14^ This implies that different normative standpoints are analysed to see whether they can be consistently held, ie, whether a so-called reflective equilibrium can be reached in relation to different combinations of these standpoints. To do so, the suggested standpoints need to be clarified, requiring enough degree of conceptual clarification. The method of reflective equilibrium can be applied in different ways. One way is to start with a well-established set of normative considerations or principles, and then analyse whether suggested applied actions are consistent with this set of principles, eg, analysing whether suggestions for restricting public coverage of uterus transplantation is consistent with established principles for priority setting, together with principles for patient and parental autonomy etc.^15^ Another way is to analyse whether two or more different standpoints can be consistently held, regardless of whether these are well-established or not. With this latter approach, we need not take a definitive stand on whether we should accept any of the suggested standpoints, but can draw conclusions as to whether we could consistently hold them. This is the approach used in this article. Specifically, this implies that RATBOT and RELCOV will be analysed in relation to the set of considerations that should guide priority setting, in an attempt to establish that regardless of what exactly this set includes, RATBOT will be supported. Following this, different explicit complementary reasons for using RELCOV that could be added to the original set of considerations will be analysed to see whether a reflective equilibrium can be reached. In doing so, RELCOV will be modified to see whether versions of RELCOV that come closer to RATBOT will better achieve a reflective equilibrium.

## Results

### Ideal Rankings, RATBOT and RELCOV

Ideally interventions ranked at level 1 are more socially valuable than those ranked at level 2 etc. With that in mind, are RATBOT and RELCOV normatively equal? In the RATBOT case all that is funded (A-C) is socially more valuable than all that is unfunded (D-E). In the RELCOV case, access is restricted for 20 patients in need of intervention B, and for 40 patients in need of intervention C. This is in order to enable access for 40 patients in need of intervention D, and 20 patients in need of intervention E. In other words - access to more socially valuable interventions is restricted in order to enable access to interventions of lesser social value. Hence, the combination of an ideal ranking list (and the values behind this) and RATBOT, is more consistent than the combination of such a ranking list with RELCOV. Expressed in other words, RELCOV does not seem to follow or adhere to the social values behind the ranking, but rather follows some other set of values. We reach a better reflective equilibrium between ideal ranking lists (and the value set behind these), and RATBOT, than between ideal ranking lists and RELCOV. I will boldly claim that this is true regardless of the coverage curve of RELCOV.

Is this the end of story, then, indicating RELCOV should not be used? Not necessarily. In the following, reasons for supporting the use of RELCOV instead of RATBOT will be explored to see if we can find an expanded set of considerations, in which ranking lists and RELCOV could be harmonised. Norheim presents a similar argument in discussing unacceptable trade-offs in universal health coverage, where central public health interventions make way for less essential interventions.^1^

### Non-ideal Ranking

Above, an ideal ranking list was assumed, ie, a list in which the background set of considerations has been consistently applied, implying that everything with a higher rank is *de facto* more socially valuable than anything with a lower rank. In a real priority setting situation, this might not (or rather, is unlikely to) be the case. There are several possible reasons for this. First, the set of considerations might be conceptually unclear.^16^ If severity and unmet needs are part of our set of considerations, how should these concepts be understood? Is there a conceptual overlap that requires to be taken into account, eg, if a need is unmet, does it also imply that the severity of the condition is unaffected, and so on? Second, there might be lack of knowledge to fully assess the different parts of our set of considerations even given a certain conceptualisation. For example, how do we measure different aspects of severity? Third, different parts of our set of considerations might be vague or even indeterminate in relation to each other. If a condition is severe but with access to moderately effective treatment, and another condition is less severe but there is an unmet need with no access to interventions, how should these be ranked in relation to each other?^17^ And fourth, the ranking might be inconsistent. We might not be meticulous enough or allow other concerns such as idiosyncratic personal or professional interests to come into play.

If we accept that these factors might influence the ranking, the ranking list might actually look like it does in Table 3. Here it is assumed that in the ideal world all A-interventions are more socially valuable than all B-interventions etc. Hence, the reason why B1 ends up at ranking 1, A4 at ranking 2 etc, depends on a combination of the above (and other possible) disturbing factors. If we got it right given our considerations, A4 should have ranking 1, B1 should have ranking 2 and so on.

**Table 3:** Distribution of Different Interventions in a Non-ideal Ranking

**Ranking**	**Interventions**
1	A1, A2, A3, B1
2	A4, B2, B3, C1
3	B4, C2, C3, D1
4	C4, D2, D3, E1
5	D4, E2, E3, E4

In this example (Table 3), the ranking is somewhat, but not totally, wrong. Most of the socially more valuable interventions are ranked above less socially valuable interventions. If this is likely to be closer to real world rankings, is this a reason to use RELCOV rather than RATBOT? Consider a developed example in Table 4^ [4] ^.

**Table 4:** A Non-ideal Ranking of Interventions Related to Patient and Cost

**Ranking**	**Interventions**	**No. of Patients in Need of Each Intervention**	**Total Number of Patients at Each Ranking Level**	**Total Cost (€)** **(2500 € per Intervention)**
1	A1, A2, A3, B1	10	40	100 000
2	A4, B2, B3, C1	10	40	100 000
3	B4, C2, C3, D1	10	40	100 000
4	C4, D2, D3, E1	10	40	100 000
5	D4, E2, E3, E4	10	40	100 000

With use of RATBOT, everything ranked 1-3 should get coverage, and everything ranked 4-5 will be rationed. Given our set of considerations and the fact that we mistakenly rank some interventions higher or lower than what this set implies, intervention D1 gets coverage and intervention C4 gets rationed, contrary to what the background set of considerations implies. It is assumed that which of the interventions at each level are ranked contrary to our set of considerations, is unknown, ie, we do not know that D1 and C4 have been prioritised wrongly.

Expressed in terms of our methodology, in this situation we have not reached a full reflective equilibrium where all the different aspects of our situation consistently harmonise with each other. Would the use of RELCOV result in a more consistent situation? Consider the use of RELCOV (following the coverage curve in Table 2), in Table 5.

**Table 5:** A Non-ideal Ranking Using a Relative Degree of Coverage

**Ranking**	**Interventions**	**No. of Patients Getting Access**	**Total Cost (€)**
1	A1, A2, A3, B1	40 of 40	100 000
2	A4, B2, B3, C1	32 of 40	80 000
3	B4, C2, C3, D1	24 of 40	60 000
4	C4, D2, D3, E1	16 of 40	40 000
5	D4, E2, E3, E4	8 of 40	20 000

That is, with luck, the 8 patients losing out on interventions at level 2 will be the ones in need of intervention C1 – the lowest ranked intervention at level 2 (from an ideal perspective). With less luck they might instead be patients in need of intervention A4 (the highest ranked intervention from an ideal perspective), who will lose out. Likewise moving down the ranking list.

To minimise the risk of ending up in the least lucky situation, one possible way is to dilute interventions so that all patients at level 2 get only 80% of the intervention, and so on. However, in many cases that solution is not feasible (eg, when it comes to surgical interventions, drug dosage etc). More importantly, if only 80% of the intervention is offered, the effect and cost-effectiveness will be different. It might also affect other relevant aspects of the intervention, which should then have had a different ranking. Moreover, with this coverage curve, at level 5, patients are still given access to interventions less important than those that patients get access to in the RATBOT case. Hence, it seems the use of RELCOV, even given non-ideal rankings, would not result in a more consistent situation, given our background set of considerations. With RATBOT, to the extent we actually have managed to get relatively more socially valuable interventions at the upper part of the ranking list, and relatively less socially valuable interventions at the lower part, relatively more socially valuable interventions will be funded than less valuable ones. Hence, even given a non-ideal ranking situation, it seems reasonable to use RATBOT rather than RELCOV (at least given the type of curve in Table 5 above, with relative coverage of all ranking levels).

A specific type of RELCOV could however be envisaged, with only relative coverage at the borderline. Consider the example in Table 6.

**Table 6:** A Non-ideal Ranking With a Relative Degree of Coverage at the Borderline

**Ranking**	**Interventions**	**No. of Patients Getting Access**	**Total Cost (€)**
1	A1, A2, A3, B1	40 of 40	100 000
2	A4, B2, B3, C1	40 of 40	100 000
3	B4, C2, C3, D1	32 of 40	80 000
4	C4, D2, D3, E1	8 of 40	20 000
5	D4, E2, E3, E4	0 of 40	0

In this case the risk of not funding socially valuable interventions at the cost of less socially valuable ones, is minimised. In a sense, RELCOV is here closing in on RATBOT. However, the problem of luck remains. That is, assuming that it is unknown which interventions we have assessed wrongly, we might end up funding the wrong interventions at level 3 and 4. In the least lucky scenario, 16 interventions in Table 6 will be wrongly funded. In the RATBOT scenario, where all interventions at level 1-3 and no interventions at level 4-5 are funded, 20 interventions will be wrongly funded. So, it seems a RELCOV approach with overlap at the borderline might, in some cases, be better than a RATBOT approach in the sense of being more consistent with our background considerations. However, we will have difficulty knowing when this is the case. If the coverage curve would have funded 30 of 40 and 10 of 40 at each side of the borderline, instead of 32 of 40 and 8 of 40, worst case scenario in RELCOV would have equalled RATBOT. Still, it might be argued that there is a chance that we fund less interventions wrongly (since we might be lucky and not end up with the worst case scenario) with RELCOV, than with RATBOT (which will give a certain outcome of 20 wrongly funded interventions).

Concluding this section, the fact that the ranking lists are not ideal could provide some reason to have a RELCOV approach with overlap just at the borderline. However, we will have difficulty knowing exactly how to set that overlap, in order to do better than with the RATBOT approach.

### Having an Opportunity to Receive Treatment

It has been suggested that one reason for using RELCOV is that all patients (regardless of condition), should at least have some opportunity to get treatment^ [5] ^. In the literature on equity in healthcare this is expressed in terms of equal opportunity. This idea might be interpreted in terms of both equal opportunity to access treatment and equal opportunity for health or quality of life (QoL).^18^

In essence, the idea of equal opportunity for health or QoL seems to take need or severity into account, if the idea is interpreted in terms of patients being entitled to levels of health equal to that of other individuals in society. Thereby the idea is likely to already be included in the set of ethical concerns guiding the priority setting above.

Let us therefore focus on the idea of equal opportunity for treatment. This idea might be interpreted as allowing all patients equal opportunity to get existing interventions, whatever the condition or treatment characteristics. This implies that all available interventions should be offered. If so, prioritising between interventions is not an option. Such an approach obviously begs the questions, and it seems more reasonable to interpret the idea of equal opportunity as a side-constraint on other ethical concerns, ie, as a formal equality constraint. Hence, two patients or groups with equally severe conditions, and similarly effective and cost-effective interventions (and similar in other ethically relevant respects), should have equal opportunity to access treatment.^19^

With such an interpretation it turns out the norm with regard to equal opportunity, would support RATBOT rather than RELCOV, given ideal ranking. With RATBOT, all patients needing interventions at a certain ranking, will either have or not have access to it, ie, equal opportunity^ [6] ^. With RELCOV, a fraction of the patients needing interventions at the same ranking, will get access, ie, unequal opportunity. If instead, the ranking is non-ideal, we have a similar situation that has been dealt with in the previous section, with some support for RELCOV at the borderline but with insecurity as to whether we have improved the situation or not.

So, is there another idea here? One such idea could be that regardless of how interventions are ranked, patients should have *some* opportunity to have access to them, though the opportunity would diminish with lower rank. RELCOV could be a way to implement such a view. Can we find a reasonable ethical rationale behind such an idea? The rationale could hardly be equity. Equity should be a central aspect of the ethical concerns, taken into account and built into our ranking. Thus, it seems unfair to give opportunity to access treatment for patients in need of less socially valuable interventions, at the cost of access to patients in need of more valuable interventions.

Another idea could be hope. That is, it is important that all patients, regardless of situation, have some hope of treatment. However, use of RELCOV would imply buying hope for patients with conditions at the lower end of the ranking, at the cost of denying patients at the high ranking end access to more socially valuable interventions, and thereby somewhat lessen hope for those in need of these interventions. Moreover, if hope is considered important enough to trump the ethically relevant concerns at the base of our ranking, it is better from the perspective of consistency to build considerations of hope into the original set of ethical and other concerns guiding the ranking in the first place.

In conclusion, it is hard to find that providing patients with the opportunity to access existing treatment is a reason to support RELCOV. On the contrary, it seems reference to opportunity to access treatment, whether for equal opportunity or hope, will support RATBOT and hence we achieve our reflective equilibrium to a greater extent in the latter than in the former case.

### Priority Setting at Group and Individual Levels

Another idea to defend the use of RELCOV, is the need to manage inconsistencies between the group and individual levels of priority setting^ [7] ^. Normally, priority setting in healthcare is done at group level, as illustrated by the examples above. This will imply that all ethical and other considerations at the base of our ranking will be about a group of patients in need of a specific intervention.

For example, to the extent that severity of condition is taken into account, it is understood as the mean or median severity of the group, and likewise effectiveness etc. Individual patients might differ from the group, implying that the interventions should have been ranked differently when offered to them. Once again this can be illustrated by the ranking list in Figure.

**Figure:**
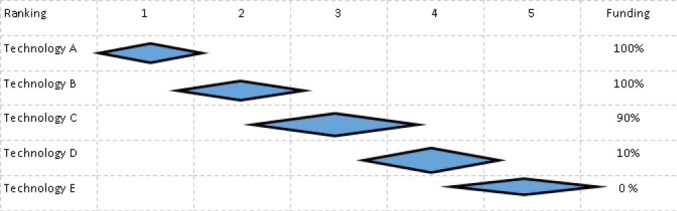
A ranking of interventions where individual patients in a patient group are distributed over more than one ranking level together with relative coverage at the borderline.

As illustrated in Figure, a patient in need of intervention D, can have a profile that would motivate a ranking 3 rather than 4, ie, for every patient group in need of a specific technology there are individual outliers in both directions that would motivate a higher or lower ranking. For example, they have a higher severity, or better effect of treatment or other features that would motivate a different ranking. Then RATBOT will block access to treatment for such patients. Would this problem be solved by use of RELCOV, or are there other ways to solve or handle it?

With RELCOV, there would be some access to treatments at all ranking levels. Access could in turn be limited only to patients that deviate from the ranking profile of their group mean, ie, that are identified at the individual level as having a higher ranking given the specific characteristics of said patients. Likewise, since in every group there will be patients and interventions with a lower rank at the individual level, they could be the ones who pay the cost at every ranking level. This seems reasonable enough.

However, first, it might be argued that RELCOV will be too indistinct as an instrument to handle this problem, and that too many resources are assigned for lower ranked interventions, just in case, with a corresponding cost for higher ranked interventions. The longer a patient group is from the borderline of what is funded with the RATBOT approach, the less likely it is that there is such great individual variation that individual patients should be ranked above the funding limit. Hence, the problem mainly arises at the borderline between what is funded and what is not with RATBOT. If there is such great variation at the individual level to motivate a RELCOV approach that assigns large amounts of resources just in case - we might question the use of ranking lists at all.

Second, to the extent that sub-groups of patients where the intervention would get a different ranking could be identified, this would preferably be done beforehand.

Third, if it is impossible to identify such sub-groups beforehand (or to judge the size of the groups and thereby the resources needed), it seems that a more limited version of RELCOV, allowing for some overlap at the borderline, would generally be a better alternative. Consider Figure again.

In this case interventions A and B are funded to 100%, intervention C is funded to 90 % implying that the patients in need of C but having a lower ranking than the rest of group l (if possible to identify), should not receive the technology, and 10% of patients at level 4 should receive funding since some of these patient (once again if identifiable), should have a higher ranking.

In conclusion, the argument from the need to distinguish between the group and individual level, could lend some support for a RELCOV approach. However, caution is needed regarding which curve of coverage to use in order to balance the different considerations in our reflective equilibrium, and it seems reasonable to use a RELCOV curve close to the RATBOT approach. This is unless there are reasons to believe that our ranking lists are far off the mark. In which case, whether adjustments could be made by looking at sub-groups to get more apt ranking lists should be explored. If this is impossible, the use of ranking lists could come into question.

### The Need to Generate Resources for Healthcare

Another argument that could support the use of RELCOV is the need to generate resources for healthcare. Such a consideration can be part of the original set of considerations to take into account when deciding on a ranking, eg, by integrating a societal perspective in the cost-effectiveness assessment taking productivity effects into account.^20^ However, due to ethical reasons this might not be viable. For example, in the Swedish context, the ethical principles guiding priority setting, explicitly claim that social situation or chronological age are not relevant considerations to take into account. This has been taken to imply that one should be careful when integrating productivity effects in the cost-effectiveness assessment – since the risk is of benefiting younger age groups with the potential to return to work, at the expense of older age groups or groups without such potential^ [8] ^. Hence, for the sake of argument, let us assume that the need to generate resources for healthcare is not part of the background set of considerations. Could RELCOV then be a way to balance this after the ranking is decided?

First, why would RELCOV be a better option than RATBOT here? Depending of course on the exact set of considerations at the bottom of our ranking, and the extent to which severity is taken into account – patients at the higher end of our ranking are likely to be in a worse state than patients at the lower end. This also, generally, implies that patients at the lower end might have a better chance to return to work after treatment, than patients at the higher end. Prioritising resources to patients at the higher end, at the expense of patients at the lower end, is therefore likely to be less beneficial to societal production than if resources are spent on patients at the lower end of the ranking. For example, patients with cancer, chronic heart failure, amyotrophic lateral (ALS) etc are likely to be highly represented at the higher end of the ranking, whilst patients with back pain, minor orthopaedic conditions, minor psychiatric illnesses etc, might be highly represented at the lower end. With interventions, the latter group might return to work, which might not be the case for the former group if the conditions are serious enough. If so, and if resources are not sufficient to cover healthcare for all technologies on the ranking list, we might need to take resources from more highly ranked alternatives to sponsor lower ranked alternatives in order to have a continuous stream of resources to distribute. This appears to be an example of applying RELCOV. Unless the healthcare system can contribute to the generation of resources for distribution, eventually even fewer patients will get access to relevant interventions. In this sense, from a systems perspective, it can be argued that applying RELCOV to our ranking lists will result in a more reasonable reflective equilibrium, all things considered, than RATBOT.

Hence, the need to generate resources for healthcare through getting people back into production could provide a reason to support the use of RELCOV.

### The Need to Get Public Support for Priority Setting

Another argument to support RELCOV could be the need to get public support for the rationing decisions made. Even if there is a general understanding by the public of the need to make rationing decisions, these decisions are more difficult to accept for those affected.^21,22^ Depending once again on the exact set of considerations used to formulate a ranking list, different patient groups will be differently represented on ranking lists following these considerations. Following the analysis in the former section, people with less severe conditions are likely to be overrepresented at the lower end of the rankings, and thus interventions for them are potentially rationed with RATBOT. Using RATBOT will imply that these patient groups will lose out on publicly financed treatment, and if decision-makers are transparent about this, they will know that they do not have any chance of getting treatment through public channels. On the other hand, with RELCOV, following a distributional curve such as the one in Table 2, all groups will have some chance of getting access to treatment. It might be easier to get public acceptance for a rationing scheme where a person knows that from whatever condition they suffer – they will have some chance of getting treatment. Hence, such a consideration will probably lend support to a RELCOV approach before a RATBOT approach. Moreover, using a RATBOT approach will generally imply that patient groups suffering from less severe conditions are less prone to get treatment in a rationing situation, all things being equal. These groups are generally in a better position to voice discontent about not receiving treatment and might thereby contribute to a negative attitude towards the healthcare sector and its priority setting. The need to generate support for priority setting in society could therefore lend some support for a RELCOV approach.

## Discussion

The above analysis shows first, if the ranking is an ideal ranking or close to an ideal ranking, RATBOT will get strongest support as the coverage scheme best in line with the ranking. However, since we might have reason to expect that, in different ways, our ranking digresses from what is ideal, we see that this could give us some reason to prefer RELCOV, if we use a coverage curve or relative distribution which allows for overlap at the borderline for what should and what should not have been covered with RATBOT. In essence, a RELCOV distribution that does not differ very much from RATBOT. This, since it was assumed that the ranking is still fairly well in line with our background set of considerations.

But what if our ranking is even further off the mark? Would the suggested RELCOV distribution exemplified in Table 2 not be a way to balance that? First, the more we get it wrong, the less value there will be in doing ranking exercises. That is, if we are that far off the mark, it might be meaningless to do ranking exercises at all – in that ranking is a way to implement the background set of considerations. Second, however, a vague or non-ideal ranking list would be combined with a curve of coverage that might enhance the ethically wrong or non-ideal ranking (depending on whether we are lucky or unlucky). Hence, it would be difficult to see RELCOV as the solution to balancing greater problems with ranking if we take our background set of considerations seriously.

It was not found that opportunity for treatment, in the sense that would support RELCOV, could possibly harmonise with our background set of considerations, since opportunity for lower ranked interventions are bought at the cost of opportunity for higher ranked interventions – in a sense self-defeating. However, the fact that the ranking is normally done at group level with a need to handle individual variations within this group, did provide some support for RELCOV. But as before, this is a blunt instrument and the more we are able to specify sub-groups and rank them according to their specific characteristics – the better the harmonisation with the rest of our considerations. If this is not possible, RELCOV at the borderline of what our resources can cover, could provide the opportunity to make adjustments at the individual level and still following the background set.

A more general reason to support RELCOV could be the need to generate resources for the healthcare system, as we saw above. Distinct from the non-ideal rankings, such a reason would probably support a more expanded version of RELCOV that not only overlaps at the borderline, but provides coverage for the groups most likely to get back into work, wherever they are on the ranking list. If we agree that the system’s ability to generate resources for distribution should be part of our reflective equilibrium, is applying RELCOV to a ranking list, without such considerations among the basic set of considerations, the best approach?

First, this seems to be a case of “double standards,” ie, there is a set of official norms and considerations to guide the ranking and then, when applying this ranking in practice, a different set of principles is introduced, because the first set is (socially) untenable. To exemplify, assume that we should consider severity of condition and cost-effectiveness in priority setting. These two factors are given internal weight so that the more severe a condition, the less cost-effective the intervention used for treatment has to be. It could turn out that what should primarily be prioritised within the system, given such considerations, has the side-effect that patients unable to return to or enter into production, get resources at the expense of less severely affected patients who can resume productivity. Over time, fewer resources are generated for the healthcare system, which eventually might affect access to treatment for those most severely affected. This ethical approach seems therefore untenable and counterproductive of its own intentions. In order to avoid this, we introduce another consideration, ie, the need to generate resources. However, this is not part of our original set of considerations for deciding a ranking, but a later add-on.

A more upright and transparent approach would be to include the full set of considerations in the original ranking – to the extent that it is possible. For example, allow productivity concerns to enter into the equation and partly guide the ranking, parallel to aspects such as severity and cost-effectiveness. One way could be to allow for productivity concerns in the cost-effectiveness assessment. But obviously it could also be done by allowing productivity effects to be factored in as yet another concern, besides severity and cost-effectiveness (or whatever list of ethical and other concerns we base our ranking on). The productivity effects could be weighted according to their considered societal importance and resource implications.

Are there any reasons to have a double standard or not be transparent and explicit about the need to generate resources for healthcare? First, it might be difficult to factor in productivity concerns in the original ranking – since productivity effects might not be transparent until the ranking is done. However, this could be solved by doing the ranking in two steps – but still have productivity concerns as part of the set of considerations. Second, there might be an ambition to uphold a more “ideal” version of priority concerns to be explicitly taken into account – perhaps for legitimacy concerns (see below). However, RELCOV is an indistinct instrument, not necessarily with the effect of targeting patient groups where the productivity effects are the greatest, or sufficient to uphold reasonable healthcare funding. An alternative to RELCOV could be more targeted approaches. That is, generally applying RATBOT, but singling out groups essential to get back into production. The needed resources should be taken from groups/interventions just above the rationing limit (patient groups who are then not candidates for getting back into production).

A potential problem by systematically and openly taking into account the ability to generate resources for healthcare is that it will, explicitly, disadvantage old age pensioners, people with certain disabilities etc, who are not, or less able to be productive in this sense (even with treatment). However, they are also likely to be disadvantaged if the resource problem in healthcare is emphasised and if they are unlucky, they might also be disadvantaged by using RELCOV as a means to generate resources. Hence, an explicit or targeted approach might also be the best option from their perspective, all things considered.

The last reason explored in the above analysis, was the need to acquire public acceptance for priority setting, at least in publicly financed and politically driven healthcare systems. Often, when arguing that there is a need for priority setting based on ethically well-founded guiding principles or aspects, transparency is viewed as essential. In cases where explicit principles are applied, these are often the result of political democratic decision-making (eg, the principles in Sweden and Norway).^3,4^ In such situations, using a RELCOV approach would be at odds with the explicit principles or aspects democratically adopted as normative, in order to make priority setting legitimate among the public. This voices once again a problematic double standard in the healthcare system. That is, if there is general public acceptance of the guiding ethical aspects or principles, and of recognising their implications when applied - these principles should be transparently applied and the public held to its word. If not, there are reasons to revise/readjust the guiding principles or aspects.

Obviously, this represents an ideal of trying to find a rationally consistent application of ethical and other considerations in different cases, not necessarily reflected in public opinion. First, as has been noted in the long philosophical debate on particularism, it might be an ideal in the sense that it seems impossible to formulate principles and aspects that will be acceptable in, or solve, all the different situations encountered. This is due to the complexity of our moral reality.^23^ Second, such an ideal rests on the acceptance of impartiality, ie, people accepting ethical principles etc. even if the application of these principles will disadvantage them in specific situations – something we might not always be psychologically prone to do.^24,25^ Given this, using the form of RELCOV, where patients at all ranking levels have a chance of some treatment, even if the chance increases with rank, might be our best option to get democratic acceptance for priority setting.

It should then be noted that if using RELCOV is required to buy public support, this is bought at the cost of not funding higher ranked treatment, thereby not reflecting the explicit (and democratically accepted) set of considerations. Following the reasoning in relation to resource generation, it can also be bought at the cost of better ways to ensure that the total bulk of resources for distribution is as large as possible. To put it bluntly, public support might then be bought at the cost of lower and less ethically justified (in terms of distributive justice) public health.

## Concluding Remarks and Implications for Policy

In this article different translations of a ranking list into resource distribution are analysed, and it is argued that they have different normative implications. Accepting that ethical considerations should guide a ranking of conditions and treatments, there are strong reasons to use rationing from the bottom when translating this ranking into resource distribution, rather than applying relative coverage, given an ideal ranking. A number of reasons could potentially support relative coverage; non-ideal ranking, enabling people to have a chance of treatment, distinguishing between the individual and group levels, and productivity concerns, could all support deviations from a strict rationing from the bottom approach. However, they still do not support a general relative coverage approach and it should be observed that they seem to support different relative coverage schemes.

The strongest support for a relative coverage approach is found in the argument of public support for priority setting, unless the public can be convinced to accept the implications of the general ethical principles and other considerations they find relevant to take into account. However, such support is bought at the cost of downplaying more central ethical considerations and possibly also at the expense of general public health.

From a policy perspective, the analysis shows that to the extent priority setting is done through ranking, it does matter how such a ranking is implemented into a coverage scheme – if the background set of considerations are taken seriously. To the extent policy makers want to be explicit and consistent in how priority setting is done, “mismatches” between this set and the coverage scheme need to be explained and given a proper rationale. The analysis shows that different rationales for digressing from RATBOT into RELCOV, do support different versions of RELCOV. Moreover, often the reasons supporting RELCOV will provide even stronger support for more targeted and explicit approaches.

## Acknowledgements

I am grateful for comments on earlier drafts of the article from colleagues at the National Center for Priority Setting in Health-Care, Linköping University, Linköping, Sweden and from Susanne Waldau at Västerbotten County Council, Sweden. I have also received valuable comments from three anonymous reviewers.

## Ethical issues

This is not an empirical study therefore ethics review is not required.

## Competing interests

Author declares that he has no competing interests.

## Author’s contribution

LS is the single author of the paper.

## Endnotes


[1] An important insight into priority setting is that different technologies should often have different priorities in relation to different conditions, due to differences in severity of the condition or effect of the treatment given the condition etc. However, for simplicity I will henceforth talk in terms of prioritising technologies (but implying that they are related to specific conditions).

[2] There might be more ideas regarding this, but in this article I will focus on these two different ideas and just claim that I think the reasoning in this article can be generalised to also cover such ideas.

[3] “Rationing” will, in this context, mean that access to a certain technology is limited for patients in need of that technology, despite being on balance effective. Rationing might imply that people get limited access but also that they get no access at all to the technology. In this context, it is mainly used to indicate no access.

[4] The available budget remains at 300 000 €.

[5] I owe this point to a comment at the Priorities 2016 conference in Birmingham.

[6] This might not be entirely true, since the budget constraint might have us draw the line within a certain ranking order.

[7] I owe this point to a comment at the Priorities 2016 conference in Birmingham.

[8] Personal communication by Douglas Lundin, chief economist at the Swedish Dental and Pharmaceutical Benefits Agency.


## Key messages

**Implications for policy makers**

The results of this theoretical analysis could benefit policy makers by:

- Showing that how priority setting and ranking of interventions are translated into coverage schemes will have implications for whether background ethical norms are implemented or not.
- Providing theoretical models and reasons to develop coverage schemes for ranking lists that are in line with the central ethical norms and other considerations of the healthcare system.
- Pointing to the fact that some coverage schemes and the associated reasons will be bought at the cost of not being able to fully implement the background ethical norms, that in essence there is an “ethical opportunity cost."

**Implications for the public**

The implications for the public are of a more indirect nature, but the coverage schemes used will obviously affect the public. It has been argued that the extent to which the public should be involved in decision-making concerning priority setting, should primarily be involvement at a policy level. Being clear over how different policies concerning coverage schemes related to the background set of ethical norms and considerations (over which they would then also have an influence), will be important input to their decisions. In communication with the public concerning different rationing decisions, it might be important to be able to point to the fact that if they also condition their acceptance on some access to lower ranked interventions – this will be bought at the cost of actually fulfilling the background set of ethical norms.
